# Cellulosic ethanol production via consolidated bioprocessing by a novel thermophilic anaerobic bacterium isolated from a Himalayan hot spring

**DOI:** 10.1186/s13068-017-0756-6

**Published:** 2017-03-21

**Authors:** Nisha Singh, Anshu S. Mathur, Deepak K. Tuli, Ravi. P. Gupta, Colin J. Barrow, Munish Puri

**Affiliations:** 1 Bioprocessing Laboratory, Centre for Chemistry and Biotechnology, Deakin University, Waurn Ponds, VIC 3217 Australia; 2 DBT-IOC Centre for Advance Bioenergy Research, Research & Development Centre, Indian Oil Corporation Limited, Sector-13, Faridabad, 121007 India; 3 Centre for Marine Bioproducts Development, Medical Biotechnology, Flinders University, Adelaide, Australia

**Keywords:** Cellulosic ethanol, Dilute acid pretreatment, Rice straw, Co-culture fermentation

## Abstract

**Background:**

Cellulose-degrading thermophilic anaerobic bacterium as a suitable host for consolidated bioprocessing (CBP) has been proposed as an economically suited platform for the production of second-generation biofuels. To recognize the overall objective of CBP, fermentation using co-culture of different cellulolytic and sugar-fermenting thermophilic anaerobic bacteria has been widely studied as an approach to achieving improved ethanol production. We assessed monoculture and co-culture fermentation of novel thermophilic anaerobic bacterium for ethanol production from real substrates under controlled conditions.

**Results:**

In this study, *Clostridium* sp. DBT-IOC-C19, a cellulose-degrading thermophilic anaerobic bacterium, was isolated from the cellulolytic enrichment cultures obtained from a Himalayan hot spring. Strain DBT-IOC-C19 exhibited a broad substrate spectrum and presented single-step conversion of various cellulosic and hemicellulosic substrates to ethanol, acetate, and lactate with ethanol being the major fermentation product. Additionally, the effect of varying cellulose concentrations on the fermentation performance of the strain was studied, indicating a maximum cellulose utilization ability of 10 g L^−1^ cellulose. Avicel degradation kinetics of the strain DBT-IOC-C19 displayed 94.6% degradation at 5 g L^−1^ and 82.74% degradation at 10 g L^−1^ avicel concentration within 96 h of fermentation. In a comparative study with *Clostridium thermocellum* DSM 1313, the ethanol and total product concentrations were higher by the newly isolated strain on pretreated rice straw at an equivalent substrate loading. Three different co-culture combinations were used on various substrates that presented two-fold yield improvement than the monoculture during batch fermentation.

**Conclusions:**

This study demonstrated the direct fermentation ability of the novel thermophilic anaerobic bacteria on various cellulosic and hemicellulosic substrates into ethanol without the aid of any exogenous enzymes, representing CBP-based fermentation approach. Here, the broad substrate utilization spectrum of isolated cellulolytic thermophilic anaerobic bacterium was shown to be of potential utility. We demonstrated that the co-culture strategy involving novel strains is efficient in improving ethanol production from real substrate.

**Electronic supplementary material:**

The online version of this article (doi:10.1186/s13068-017-0756-6) contains supplementary material, which is available to authorized users.

## Background

Cellulosic ethanol represents an important sustainable alternative to support the global demand for renewable liquid transportation fuels [1]. Lignocellulosic biomass (containing 50–70% fermentable carbohydrates in the form of cellulose and hemicellulose), is an attractive feedstock for the production of cellulosic ethanol, due to its abundant availability, low cost and possible environmental benefits, as it does not compete directly with food crops [2, 3]. However, the conversion of cellulose and hemicellulose to fermentable sugars is a rate-limiting step due to its highly recalcitrant nature [4]. Thus, a pretreatment step is required in addition to the exogenous addition of costly cellulolytic enzymes prior to fermentation, which significantly increases the cost of bioprocessing [4, 5].

Consolidated bioprocessing (CBP) accomplishes production of cellulolytic enzymes, hydrolysis of lignocellulose and fermentation of resulting sugars (C_5_ and C_6_) to ethanol, or other valuable products, in a single vessel or reactor with low process complexity. CBP is therefore an economical approach for the production of second-generation biofuel [2]. This single-step conversion technology is based on a candidate CBP microbe or group of microbes having combined hydrolysis and fermentation ability [4, 6]. Industrially relevant targets that determine the cost effectiveness of CBP-based cellulosic ethanol production are a yield of >90% of the theoretical maximum, titer of >40 g L^−1^, and productivity of ~1 g L^−1^ h^−1^ [7]. However, no single candidate microorganism with this level of potential is known so far [8]. CBP at high temperature could be more advantageous than at lower temperature by offering improved catalysis, lowered energy requirements, minimized contamination risk, and maximized cost effectiveness [9, 10]. Cellulose and/hemicellulose-fermenting thermophilic anaerobic bacteria particularly those belonging to the genus *Clostridium* and *Caldicellulosiruptor* have potential for CBP due to their efficiency in the conversion of cellulosic substrates into ethanol [11, 12].

Central to this approach is *Clostridium thermocellum,* a thermophilic, strictly anaerobic and Gram-positive bacterium, which is an excellent CBP candidate due to its remarkable cellulose solubilization ability [8, 13]. *Clostridium thermocellum* is known for its complex machinery of lignocellulolytic enzymes called the “cellulosomes,” providing a capability to grow well on crystalline cellulose [14, 15] with comparable hydrolysis performance to commercially available cellulolytic enzymes [12, 16–18].

Despite this high cellulolytic potential, *C. thermocellum* catabolizes pyruvate via a mixed acid fermentation pathway that involves formation of other products such as lactate, acetate, formate, hydrogen, and excreted amino acids, in addition to ethanol, thus resulting in lower ethanol yields [19]. Also, an inability to utilize C5 sugar in conjunction with relatively low ethanol tolerance is the major disadvantage associated with *C. thermocellum*-based fermentation of lignocellulosic biomass [8, 20]. Applying a co-culture of *C. thermocellum* with other ethanologenic thermophilic anaerobic bacteria can improve substrate utilization and product yield due to synergistic effects [21, 22].

A number of *C. thermocellum* strains have been isolated from diverse natural and man-made environments, which presented significant disparity in their physiological characteristics, substrate utilization, cellulosomal subunit composition, and cellulose degradation potential [23–31]. Interestingly, the type strain of this bacterium ATCC 27405 is an efficient cellulose degrader. However, few isolated strains with higher ethanol production [27] and cellulosome hydrolytic potential [29] have been identified.

Considering this, a broad screening of diverse environment for cellulose-degrading thermophilic anaerobic bacteria is important for the identification of novel strains with efficient lignocellulose degradation ability for strengthening the viability of a CBP approach.

With this background, the aim of the current study was to identify novel strains of cellulose-degrading thermophilic anaerobic bacteria that are capable of direct lignocellulose conversion to ethanol. A Himalayan thermal hot spring site was chosen as an extreme location for this study. The site was a remote site and so there was limited chance for human modification of the natural microbial flora. To the best of our knowledge, this is the first study presenting the isolation and characterization of cellulose fermenting and ethanol-producing anaerobic bacteria from a Himalayan hot spring site.

Most of our current understanding of ethanol fermentation employing CBP candidate microbes is based on fermentation of model cellulosic and hemicellulosic substrates (i.e., cellulose and xylan) [15, 32–34]. In comparison, limited studies have focused on real substrate-based ethanol fermentation [35–37]. Achieving improved ethanol production from the native lignocellulose biomass is the ultimate goal for the sustainable CBP.

Rice straw biomass (RSB) is one of the most abundant lignocellulosic waste materials and could potentially produce nearly 205-billion-liter ethanol per year [38, 39]. In tropical countries such as India, huge amount of rice straw biomass is generated annually, but a large portion was burnt in the field resulting in environmental pollution [3]. Considering its high holocellulose content (approximate 55–59%), rice straw biomass was used in this study as a low-cost renewable feedstock for cellulosic ethanol production. Although pretreatment of biomass is an energy-intensive and expensive step for industrial production of cellulosic ethanol [40], dilute acid pretreatment was performed in this study. To date, no information is available for testing the feasibility of consolidated bioprocessing for cellulosic ethanol production employing combination of thermophilic anaerobic bacteria for direct microbial fermentation of rice straw biomass. The co-culture fermentation performance of newly isolated *Clostridium* sp. strain DBT-IOC-C19, with hemicellulose and sugar-fermenting bacteria, was investigated. Rice straw biomass has excellent potential as a substrate for cellulosic ethanol production by the new isolates in a co-culture.

## Results and discussion

### Enrichment, isolation, and phylogeny of the cellulose-degrading thermophilic anaerobic bacteria

Thermal hot water samples collected from various locations of the Puga hot spring were used as a source of the new cellulolytic strains. The positive cellulolytic enrichment cultures that produced ethanol from filter paper as substrate were transferred on the same enrichment medium with deacetylated dilute acid-pretreated rice straw, to stringently select cultures capable of performing direct conversion of lignocellulosic biomass to ethanol. The final choice of enrichment culture, producing ethanol as the major soluble end products, is shown in Additional file 1: Table S1, which also presented the amount of other soluble metabolites. Higher ethanol and total soluble metabolite concentrations were used as initial screening criteria to purify the isolates best suited for cellulosic ethanol production. After 48–96 h of incubation, several bottles showed positive growth in the form of turbidity. Single colonies from selected enrichment cultures displayed clear zones of cellulose solubilization indicating cellulose degradation potential. These colonies were picked with a sterile inoculation loop and sub-cultured on fresh liquid medium (M) with cellulose and cellobiose as the carbon source, and this procedure was repeated several times. During the multiple rounds of purification, some isolates lost their ability to degrade cellulose; thus, the procedure was repeated until stable cellulolytic colony forms were predominant. The obtained pure colonies were round, white to pale yellow in color, deep agar as well as in surface forms.

Nineteen cellulose-degrading thermophilic anaerobic bacteria, designated as strains DBT-IOC-C1 to DBT-IOC-C19, were obtained as pure stable cultures and compared on the basis of fermentation profiling using crystalline cellulose as substrate (Additional file 1: Table S2). Under suboptimal conditions, three strains referred as strain DBT-IOC-C2, strain DBT-IOC-C15, and strain DBT-IOC-C19, displayed the higher ethanol productivity and so were chosen for further investigation. The selected isolates differed from each other with respect to fermentation profiling, substrate spectrum, and each produced a differential quantity of yellow affinity substance (YAS) during growth on cellulose. The positive effect of this YAS on adsorption and binding of cellulolytic complex of anaerobic bacteria during growth on crystalline cellulosic substrates is well known [41, 42].

**Fig. 1:**
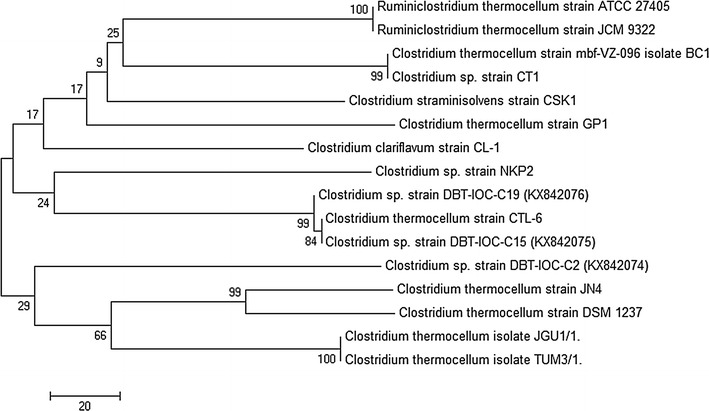
Phylogeny of isolated cellulose-degrading thermophilic anaerobic bacteria based on 16S rRNA gene sequence analysis. The percentage of replicate trees in which the associated taxa clustered together in the bootstrap test (1000 replicates) is shown next to the branches [86]. The evolutionary distances were computed using the Maximum Composite Likelihood method [87] and are in the units of the number of base substitutions per site. All positions containing gaps and missing data were eliminated. There were a total of 1392 positions in the final dataset. Evolutionary analyses were conducted in MEGA7 [85]. Gene bank accession numbers of the isolated strains are shown in* parentheses*

An analysis of the 16S rRNA gene sequence for each of these three strains revealed homology with different members of the genus *Clostridium,* and their evolutionary history is presented as a phylogenetic tree in Fig. 1. The genus *Clostridium* comprises several solventogenic and thermophilic members that are capable of performing cellulose and hemicellulose fermentation to valuable products such as ethanol and hydrogen [8]. The closest relative to all three isolates was *C. thermocellum* ATCC 27405^T^ [30], with 16S rRNA gene sequence identity ranging from 99.79% (strain DBT-IOC-C19) to 99.86% (Strain DBT-IOC-C2 and DBT-IOC-C15). The type strain of *C. thermocellum* ATCC 27405 is the most exploited member of this genus and under investigation for ethanol production via a CBP approach. Several *C. thermocellum* strains isolated from a variety of habitat have been shown in Table 1.

**Table 1:** Comparison of different *Clostridium* strains as reported in the literature for ethanol production

*Clostridium* strain	Substrate (g L^−1^)	Ethanol (mM)	Acetate (mM)	Lactate (mM)	Time (h)	Reference
ATCC 27405	Avicel 5	18.0	13.8	ND	–	[55]
	Avicel 10.3	37.0	46.0	6.0	50	[56]
DSM 1313	Avicel 5	15.2	18.3	0.6	–	[57]
	Avicel 19.5	28.7	45.6	27.6	100	[58]
S14	MCC type 20 10	41.2	61.9	8.2	72	[14]
CS7	Milled Whatman No. 1 filter paper 3	17.1	5.3	ND	20	[26]
CS8	Milled Whatman No. 1 filter paper 3	18.0	7.2	ND	20	[26]
JN4	Avicel 5	6.2	7.6	13	60	[59]
YS	Avicel 20	36.0	12.0	ND	–	[27]
ATCC 35609	MN300 8	31.2	22.5	ND	120	[60]
JW20	Avicel 10	25.4	13.0	9.4	16	[28]
YM4	Avicel 10	44.7	26.5	20.4	96	[61]
BC1	Whatman No. 1 filter paper 10	10	2	ND	144	[40]
DBT-IOC-C19	Avicel 10	32.55	18.67	5.14	96	This study

*ND* not detected, metabolite yield reported as g L^−1^ converted to millimolar using a molecular weight of 46.07 g mol^−1^ for ethanol, 60.05 g mol^−1^ for acetate, and 90.08 g mol^−1^ for lactate

### Growth optimization and metabolic properties

All three strains were able to grow only in a pre-reduced medium. Severe growth inhibition was observed in the presence of oxygen, suggesting the obligatory anaerobic nature of the isolates. Based on optical density measurement, maximum growth by *Clostridium* sp. strain DBT-IOC-C2 was achieved at pH 7.0. *Clostridium* sp. strain DBT-IOC-C15 and *Clostridium* sp. strain DBT-IOC-C19 exhibited maximum growth at slightly alkaline pH values between 7 and 8. Therefore, pH 7.5 was selected as the optimal pH for both these strains (Additional file 1: Figure S1). The strain DBT-IOC-C15 was able to grow over a wide temperature range from 55 to 65 °C with optimal temperature being 55 °C. *Clostridium* sp. strain DBT-IOC-C19 and strain DBT-IOC-C2 grew optimally at 60 °C (Additional file 1: Figure S1). The temperature and pH ranges observed here are in line with the growth optima observed for many *C. thermocellum* strains reported previously where a temperature growth range of 55–70 °C and an optimal pH close to neutral was optimal [25, 30, 43]. However, some strains with growth optima near acidic [24] and alkaline [29] pH conditions were also observed.

By comparing the cellulose fermentation profile of three selected cellulose-degrading isolates on ‘Minimal media for thermophilic *Clostridia*’ (MTC), these strains presented significant differences in ethanol production as shown by ANOVA (Additional file 1: Figure S2). All these strains produced yellow affinity substrate during growth and the main soluble metabolic end products were ethanol, acetate, and lactate. However, the amount of lactate produced by strain DBT-IOC-C2 and strain DBT-IOC-C19 was distinctly higher than the type strain of *C. thermocellum* ATCC 27405 [44]. Strain DBT-IOC-C19 displayed the most efficient degradation with the highest corresponding total soluble metabolite concentration (56.25 mM) and ethanol concentration (34.33 mM), compared to the other two strains tested and was therefore selected for the further studies.

The characteristic ability of thermophilic anaerobic bacteria to utilize a broad range of substrates is the prime reason for their consideration for consolidated bioprocessing [10]. The efficacy of these three strains to utilize a range of substrates under respective optimal growth conditions was evidenced by turbidity, product formation, and a pH drop during growth. In all cases, major soluble metabolites were ethanol, acetate, and lactate (data not shown). All new isolates grew on cellulose, carboxymethyl cellulose, xylan, filter paper, and other complex polysaccharides tested in this study, but the capability to ferment simple sugars varied between these strains (Additional file 1: Table S3). The following carbohydrates were not utilized by any of the isolate: mannose, fructose, maltose, lactose, sucrose, soluble starch, and xylose. Interestingly, the type strain ATCC 27405 can ferment fructose, suggesting differences in the metabolic behavior of new isolates compared with this type strain [24]. The new strains were found to ferment cellulose and cellobiose faster than other substrates, a characteristic similar to previously described *C. thermocellum* strains [23, 24, 45]. None of the strains were able to utilize xylose as the sole carbon and energy source, even after repeated adaptation, which was also observed for related type strain. Overall, these novel strains were found to have some similarities to the type strain in terms of optimal growth conditions (particularly strain DBT-IOC-C2), yellow affinity substance production, and no growth on xylose.

### Kinetics of crystalline cellulose degradation by strain DBT-IOC-C19

**Fig. 2:**
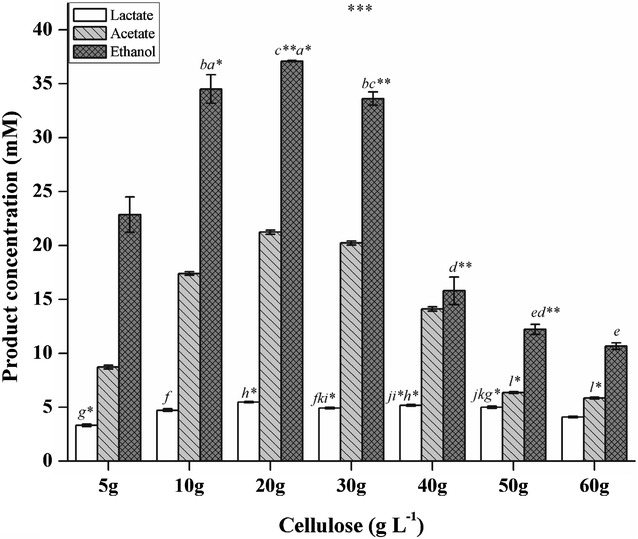
Fermentation products of *Clostridium* sp. DBT-IOC-C19 at different cellulose concentrations. The specific rate of product formation by strain DBT-IOC-C19 as a function of various crystalline cellulose concentrations measured at 60 °C (initial pH 7.5) after 96 h of batch fermentation. Specific product formation was monitored by measuring pH, acetate, lactate, and ethanol concentrations. Data are presented as average of triplicate cultures with *error bars* (±) showing standard deviations. All metabolite concentrations are different with a statistical significance [ANOVA Tukey’s test, ***p < 0.001, ** 0.01 > *p* > 0.001, and * 0.05 > *p* > 0.01]. The concentrations that are not significantly different from each other in treatment condition described are labeled with same *lowercase letters* associated with a level of significance in the* figure*

It is evident from some previous studies that initial cellulose concentration plays an important role in ethanol production rate, cellulose conversion efficacy, and metabolic flux distribution during fermentation by thermophilic *Clostridia* [15, 28, 46, 47]. A similar effect was also observed during ethanol fermentation by strain DBT-IOC-C19, with both cellulose conversion (%) and ethanol concentration significantly affected when subjected to increasing cellulose concentration as shown by ANOVA (Additional file 1: Table S4A). In this work, the substrate concentration and incubation time for the strain DBT-IOC-C19 during fermentation was optimized. *Clostridium* sp. strain DBT-IOC-C19 exhibited degradation of cellulose at seven concentrations of Avicel PH-101; 5,10, 20, 30, 40, 50, and 60 g L^−1^, as shown in Fig. 2. Analysis of the end products revealed that ethanol was produced as the major soluble metabolic end product followed by acetate and lactate, under the experimental conditions used in this study (Additional file 1: Table S4A). Other end products included hydrogen and carbon dioxide (data not shown). Similar to this observation, production of ethanol, acetate, lactate, hydrogen, and carbon dioxide by different strains of *C. thermocellum* was reported in some previous studies [23, 25, 29, 48]. Interestingly formate was also reported as a metabolic end product for the type strain ATCC 27405 [49, 50]. However, formate production by strain DBT-IOC-C19 was not evident on any of the substrate tested in this study.

**Fig. 3:**
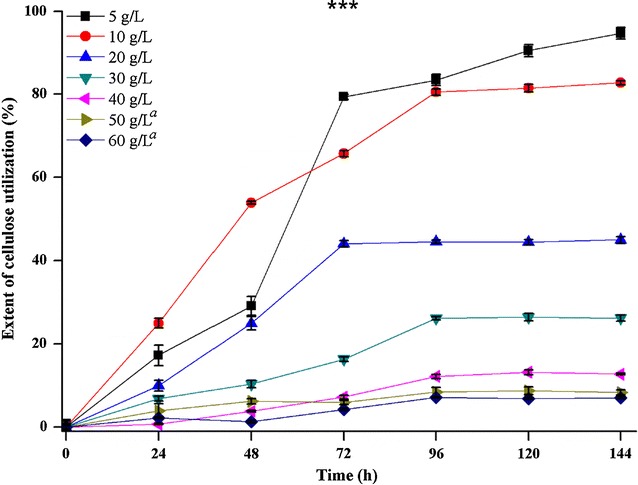
Extent of cellulose utilization by *Clostridium* sp. DBT-IOC-C19 on different avicel concentrations. Time course of crystalline cellulose utilization at various concentrations within 144 h of batch culture of strain DBT-IOC-C19, residual cellulose concentration was determined by dry weight basis. Each data value represents average with *error bars* (±) showing standard deviations calculated from triplicate fermentations. The percentage of cellulose conversion at different cellulose concentrations are different with a statistical significance [ANOVA Tukey’s test, ***p < 0.001]. The concentrations that are not significantly different from each other in treatment condition described are labeled with same *lowercase letters* in the *figure* legends

A 100% consumption of crystalline cellulose by strain DBT-IOC-C19 was not observed at any of the initial cellulose concentration tested even after a prolonged incubation time of 144 h (Fig. 3). It was observed that about 94.6% degradation was achieved at relatively low initial cellulose concentration of 5 g L^−1^ after at least 144 h of fermentation which was dropped to 7% at the highest cellulose concentration (60 g L^−1^) tested. The ethanol concentration decreased continuously from 37.09 to 10.67 mM beyond 123 mM (20 g L^−1^) initial cellulose concentration (Additional file 1: Table S4A). The cellulose conversion observed here at 1% concentration was comparable to ~95% conversion observed during cellulose fermentation by strain ATCC 27405 at equivalent cellulose concentration [51], showing the cellulolytic potential of the new isolate.

The maximum ethanol concentration (37 mM) and total soluble metabolite concentration (63.82 mM) were achieved at 20 g L^−1^ initial cellulose concentration (Fig. 2). However, at this concentration, approximately 50% of the cellulose remained unutilized and the fermentation rate was similar to what achieved at 10 g L^−1^ initial cellulose concentration, indicating that strain DBT-IOC-C19 possesses maximum avicel utilization ability of 10 g L^−1^ (i.e., 61.67 mM of glucose equivalents). This variation in cellulose utilization during the course of fermentation was attributed to decrease in the initial pH 7.5 to pH 5.70, below which the strain encountered severe inhibition of growth.

During the growth on higher cellulose concentrations, a gradual decrease in both cellulose consumption and total product concentration was more pronounced in comparison to the lower substrate concentration (Fig. 3). A similar effect was reported for other *C. thermocellum* strains in the previous reports [31, 46, 51, 52], suggesting the limited substrate tolerance of thermophilic *Clostridia.* In fact, at cellulose concentration higher than 20 g L^−1^, the amount of cellulose utilization was restricted to nearly 5 g L^−1^ only. While at 40, 50, and 60 g L^−1^ cellulose concentration nearly 87.22, 91.21, and 92.4% of initial cellulose, respectively, remain unutilized (Fig. 3; Additional file 1: Table S4B). Interestingly, the formation of lactic acid remains unaffected even at increasing cellulose concentration and was similar at all the initial cellulose concentrations tested. A limited accumulation of soluble sugars was observed at higher substrate concentration only after growth decreased (data not presented). Since the initial crystalline cellulose concentration of 10 g L^−1^ resulted in the most efficient ethanol fermentation, the kinetics of cellulose consumption at 10 g L^−1^ was characterized in more detail to get a better understanding of the underlying process. Lower ethanol yield and incomplete substrate utilization at higher cellulose concentration can be attributed to poor mass transfer and accumulation of reducing sugars in the medium. However, subsequent residual cellulose at lower substrate concentration was related to the acidification of medium mainly due to lactic acid production and growth suppression. These results are in agreement with the previous studies where a strong correlation between the growth of the bacteria and increasing cellulose concentration was suggested [53]. Islam and co-workers have suggested an improved fermentation performance by *C. thermocellum* DSM 1237 under high cellulose concentration by varying the nutrient composition of the medium and a 2.3-fold increment in the yield was reported [54].

**Fig. 4:**
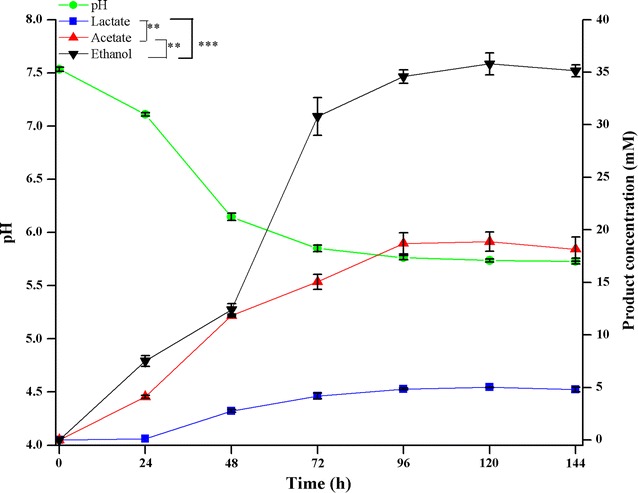
Kinetics of crystalline cellulose consumption by *Clostridium* sp. DBT-IOC-C19. Kinetics of crystalline cellulose (10 g L^−1^ avicel) consumption and metabolite concentrations obtained within 144 h of batch culture of strain DBT-IOC-C19 as a function of initial cellulose concentration. Fermentation of crystalline cellulose was monitored by measuring pH, acetate, lactate, ethanol, and residual cellulose concentration was determined by dry weight basis. Each data value represents average with *error bars* (±) showing standard deviations calculated from triplicate fermentations. All metabolite concentrations are different with a statistical significance [ANOVA Tukey’s test, ***p < 0.001, ** 0.01 > *p* > 0.001, and * 0.05 > *p* > 0.01], calculated at 144 h

The maximum rate of cellulose degradation was measured in terms of soluble metabolites produced in parallel during the course of fermentation. As shown in Fig. 4, fermentation end products such as ethanol, acetate, and lactate as well as cellulose consumption increased during the first 96 h of incubation and then began to slow down with the drop in pH from 7.5 to 5.76. At this concentration, 35 mM ethanol, 18.15 mM acetate, and 4.88 mM lactate were produced (Additional file 1: Table S4C). Cellulose was also consumed mainly within initial 96 h (80.44% consumption) and only a small increment (2.31%) in consumption was observed until 144 h of fermentation. Ethanol (30.77 mM) was mainly produced within 72 h, after which its concentration increased gradually reaching a maximum concentration (35.79 mM) at 120 h. In contrast, acetate and lactate concentrations reached 18.15 and 4.88 mM, respectively, during initial 96 h and remained constant thereafter. A slight decrease in lactate and acetate production at 144 h of fermentation can be explained by their probable utilization as a substrate for gas production by the strain DBT-IOC-C19 (Fig. 4). According to these results, the strain DBT-IOC-C19 achieved maximum ethanol and total product formation at 96 h of fermentation. Thus, duration of fermentation for 96 h was selected for conducting co-culturing studies.

### Comparative fermentation characteristics on lignocellulosic substrates

A higher ethanol yield and improved cellulose conversion were evident by the dynamics of crystalline cellulose degradation by strain DBT-IOC-C19 as shown above. However, the significance of this yield needs to be compared with other cellulose-degrading thermoanaerobes. Therefore, fermentation studies have been carried out using the potential CBP candidate bacteria, *C. thermocellum* DSM 1313, under the experimental conditions described in this study. Strain DSM 1313 was selected due to its high phylogenetic similarities (99%) to strains DBT-IOC-C19 (Fig. 1), the most thoroughly described CBP candidate [34, 47, 55, 56].

**Fig. 5:**
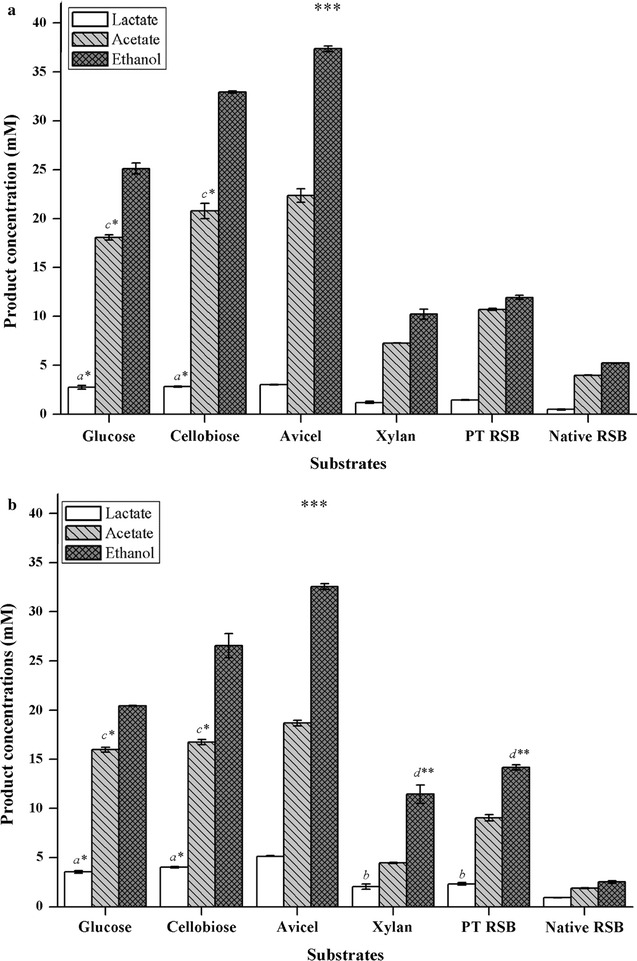
Comparative fermentation profile of *Clostridium* sp. DBT-IOC-C19 and *Clostridium thermocellum* DSM 1313 on various substrates. Comparative production of fermentation end products by (**a**) *Clostridium thermocellum* DSM 1313 and (**b**) *Clostridium* sp. strain DBT-IOC-C19 on various substrates loaded at an equivalent concentration (10 g L^−1^) expressed as xylose and glucose equivalents. Batch fermentations were maintained at 60 °C with initial pH 7.5 for strain DBT-IOC-C19 and pH 7.0 for strain DSM 1313 for 24–96 h. Progress in fermentation was monitored by measuring decrease in pH, acetate, lactate, and ethanol concentrations. Data are presented as average with *error bars* (±) showing standard deviations calculated from triplicate fermentations. The metabolite concentrations at different substrates varied with a statistical significance [ANOVA Tukey’s test, ***p < 0.001, ** 0.01 > *p* > 0.001, and * 0.05 > *p* > 0.01]. The concentrations that are not significantly different from each other in treatment condition described are labeled with same *lowercase letters* associated with a level of significance in the figure. *PT* pretreated, *RSB* rice straw biomass

The comparative fermentation performance of the novel strain DBT-IOC-C19 and strain DSM 1313 (reference strain) on various cellulosic and non-cellulosic substrates including complex carbohydrates (i.e., crystalline cellulose and xylan) and simple sugars (i.e., cellobiose and glucose) was assessed. In general, ethanol, acetate, and lactate were produced as the main soluble fermentation products on all the substrates tested, as shown in Fig. 5. However, depending upon the substrate, a variation in end product distribution was observed.

On the basis of our previous observation, an initial substrate loading of 1% (w/v) Avicel PH101 (i.e., 61.67 mM glucose equivalents) and a fermentation period of 96 h were shown to be the most suited for performing fermentation in a closed system without pH control. Therefore, an equivalent substrate loading expressed as hexose/pentose equivalents of the different substrates tested was applied for studying the fermentation profile of both the strains.

Fermentation end products on glucose and cellobiose were analyzed within 24 h of incubation. Growth and product formation were much faster on these substrates than on crystalline cellulose. During glucose fermentation, the ethanol concentration produced by strain DSM 1313 (25.12 mM) was higher than the concentration achieved by strain DBT-IOC-C19 (20.42 mM) (Fig. 5). Likewise, during the growth on cellobiose, ethanol yields were 32.93 mM and 26.55 mM, for the strains DSM 1313 and DBT-IOC-C19, respectively (Additional file 1: Table S5).

During the fermentation of Avicel PH-101, strain DBT-IOC-C19 produced 5.14 mM lactate, 18.67 mM acetate, and 32.55 mM ethanol and reference strain DSM 1313 produced 3.03 mM lactate, 22.35 mM acetate, and 37.35 mM ethanol, indicating that the total soluble metabolite concentration of the newly isolated strain was comparable to the concentration achieved by the reference strain (Additional file 1: Table S5). In contrast, during hemicellulose fermentation, the ethanol concentration by strain DSM 1313 was 10.23 mM which was slightly lower than the ethanol concentration achieved by strain DBT-IOC-C19 (11.45 mM). However, the total soluble metabolite concentration achieved by strain DSM 1313 (18.72 mM) was higher than that of strain DBT-IOC-C19 (17.95 mM) (Additional file 1: Table S5).

### Rice straw fermentation for ethanol production

From above studies, the potential of strain DBT-IOC-C19 to metabolize model polysaccharides was clear. However, further studies on real lignocellulosic substrates are essential to determine its relative importance as a potential CBP candidate for ethanol production. So far, single-step ethanol production involving different *C. thermocellum* strains has been attempted on a variety of complex lignocellulosic material such as corn stalk [57], corn cobs [58], corn stover [36], switchgrass [37, 59, 60], *Populus* [35, 61, 62], sugarcane bagasse [63–66], and wheat straw [67]. However, in order to investigate this ability for strain DBT-IOC-C19 and DSM 1313, rice straw biomass in native and pretreated forms was used and their fermentation performance was compared. To the best of our knowledge, this is the first study reporting the direct microbial fermentation of RSB for cellulosic ethanol production.

The native rice straw used for the production of pretreated biomass contained 36.9% glucan, 20% xylan, 3.5% arabinan, 13.4% lignin, 7.3% ash, 1.1% acetic acid, and 17.8% extractives as determined by the NREL method [68, 69]. To avoid misinterpretation of the results due to the soluble sugars present in the native biomass, rice straw was washed extensively with water at 60 °C (the growth temperature of *Clostridium* sp. strain DBT-IOC-C19) for 24 h and the collected dried material was used as the sole carbon source for the fermentation studies throughout this experiment. After dilute sulfuric acid pretreatment, the glucan and xylan contents of rice straw were changed to 70.6 and 4.2%, respectively, as determined by composition analysis. Therefore, medium with 14.16 g L^−1^ of washed pretreated rice straw (after moisture correction) contained about 61.67 mM glucose equivalents. In this study, only glucan content of both these substrates was taken into consideration for determining the substrate loading.

As illustrated in Fig. 5a, b, strain DSM 1313 and DBT-IOC-C19 grew and utilized pretreated as well as native rice straw and produced ethanol, acetate, and lactate as the main soluble metabolic end products under their respective optimized growth conditions (Additional file 1: Table S5). Ethanol production by both strains was found to be different when subjected to rice straw fermentation, but statistically significant as shown by ANOVA (Fig. 5). At the end of fermentation, strain DBT-IOC-C19 produced 2.31 mM lactate, 9.05 mM acetate, and 14.15 mM ethanol and strain DSM 1313 produced 1.45 mM lactate, 10.71 mM acetate, and 11.93 mM ethanol, indicating that the total soluble metabolite concentration produced by newly isolated strain was marginally higher than the reference strain. The higher lactate production by strain DBT-IOC-C19 suggested a metabolic shift in the fermentation pathway during the growth on pretreated rice straw which is suspected to be the primary reason for its higher total product concentration than DSM 1313.

### Co-culture fermentation performance

Combining different microbial strains to with synergistic characteristic provides a potential for higher ethanol production levels and efficiency, particularly when degradation of complex substrates is involved [8, 21]. In recent years, substantial effort has been carried out to increase ethanol and hydrogen production by applying co-culture of cellulolytic and non-cellulolytic thermoanaerobes, resulting in increased ethanol yield due to their symbiotic behavior offering exchange of metabolites, improved stability, and reduced lag phase. [34, 70–73]. However, in comparison to monocultures, the number of co-culture studies involving combination of pure cultures of anaerobic bacteria is limited, in particular with regard to direct fermentation of lignocellulosic substrates [26, 58, 74, 75].

**Fig. 6:**
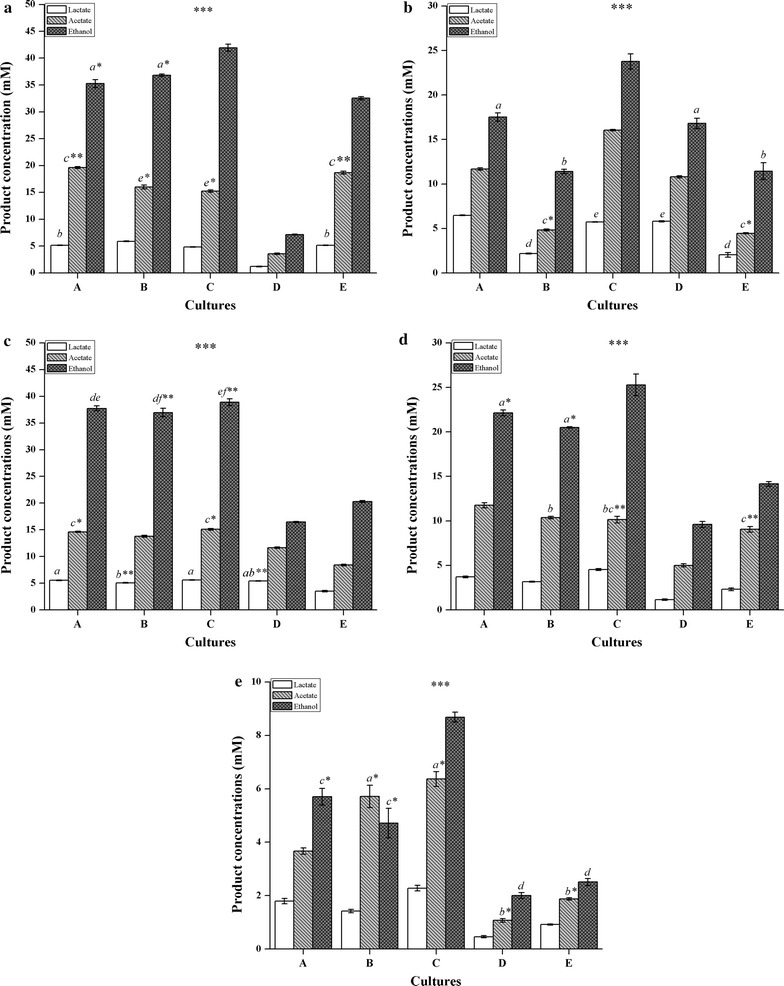
Growth and fermentation products on various substrates by monoculture and co-culture combinations. Cellulolytic and fermenting strains; *A* co-culture of cellulolytic *Clostridium* sp. strain DBT-IOC-C19 and the xylanolytic *Clostridium* sp. strain DBT-IOC-DC21, *B* co-culture of cellulolytic *Clostridium* sp. strain DBT-IOC-C19 and non-cellulolytic (fermenting) strain *Thermoanaerobacter* sp. strain DBT-IOC-X2, *C* co-culture of cellulolytic *Clostridium* sp. strain DBT-IOC-C19 and xylanolytic *Clostridium* sp. strain DBT-IOC-DC21 and fermenting strain *Thermoanaerobacter* sp. strain DBT-IOC-X2, *D* monoculture xylanolytic *Clostridium* sp. strain DBT-IOC-DC21, and *E* monoculture cellulolytic *Clostridium* sp. strain DBT-IOC-C19 were grown on various substrates in serum bottles without pH control at 60 °C. Each data point represents average with *error bars* (±) showing standard deviations calculated from samples collected from triplicate experiments. The metabolite concentrations produced by different mono and co-culture combinations varied with a statistical significance [ANOVA Tukey’s test, ***p < 0.001, ** 0.01 > *p* > 0.001 and * 0.05 > *p* > 0.01]. The concentrations that are not significantly different from each other in treatment condition described are labeled with same *lowercase letters* associated with a level of significance in the figure [Substrates for fermentation: **a** avicel, **b** xylan, **c** Mixture of avicel and xylan, **d** pretreated rice straw biomass, **e** native rice straw biomass]

In order to improve ethanol production, strain DBT-IOC-C19 was incubated with two non-cellulolytic strains, under sterile conditions to establish their co-cultures. Batch fermentation of three different combinations of novel strains, strain DBT-IOC-C19 (cellulose-degrading), strain DBT-IOC-DC21 (xylan-degrading), and strain DBT-IOC-X2 (sugar-fermenting), was carried out on various substrates and their ethanol production potential is presented in Fig. 6. The co-culture combinations (C19 + X2, C19 + DC21, and C19 + DC21 + X2) were tested on cellulose, xylan, a mixture of cellulose and xylan (to mimic biomass), pretreated rice straw and washed native rice straw (Additional file 1: Table S6).

The sugar-fermenting strain DBT-IOC-X2 could not utilize complex polysaccharides due to its inability to grow directly on these substrates. However during co-culturing, enzymatic breakdown of the complex substrates by cellulolytic strains generated soluble sugars which served as a carbon source for the strain DBT-IOC-X2. In turn, the removal of hydrolytic products in co-culture might have facilitated enzymatic degradation by cellulolytic strain. Ethanol, acetate, and lactate were the main soluble metabolic end products produced by the tested co-cultures (Fig. 6) but depending upon the substrates and culture combinations, variation in the metabolic product distribution was observed. These differences in the product formation could be caused by the more rapid consumption of the substrates by the co-cultures; however, the fermentation products were analyzed at 96 h. Here the predominant effect of various co-culture combinations and substrates on ethanol production was found to be statistically significant according to ANOVA (Fig. 6).

During co-culture fermentation, the ethanol yield from crystalline cellulose was increased from 32.55 mM for strain DBT-IOC-C19 and 7.14 mM for strain DBT-IOC-DC21 to 35.26 mM using their co-culture. When a sugar-fermenting strain DBT-IOC-X2 was employed in this combination, the ethanol yield was increased to 41.94 mM, suggesting that this combination of strains is highly efficient in converting crystalline cellulose to ethanol. Similar to ethanol, an improvement in acetate and lactate production was also observed by the co-cultures (Fig. 6a).

For hemicellulose fermentation, an improvement in ethanol concentration from 11.45 mM for strain DBT-IOC-C19 to 23.76 mM for the co-culture C19 + DC21 + X2 was observed on xylan as a substrate that represents almost two times improvement in ethanol yield (Fig. 6b). This result emphasizes the significance of the co-cultures in the bioconversion of lignocellulosic biomass rich in xylan content.

In the simultaneously performed fermentation on a mixture of cellulose and xylan, the ethanol yield was increased from 20.29 mM for strain DBT-IOC-C19 and 16.46 mM for strain DBT-IOC-DC21, to ~38 mM from the three combinations tested. The total soluble metabolite concentration of 32 mM was also increased to a maximum of 59 mM by the best combination of co-culture involving all the three strains together (Fig. 6c). This result also suggests the apparent improvement of hemicellulose fermentation performance by the strain DBT-IOC-C19 due to the combined effect of xylan-fermenting strain.

In this study, co-culturing offered a promising way to improve ethanol production from cellulose and hemicellulose. The yield improvement by co-cultures represented the synergistic action of the cellulolytic and sugar-fermenting anaerobic bacteria. Such synergism is feasible in microbial consortia growing in natural environment [8].

The favorable effect of co-culturing was recently demonstrated by Xu et al. who showed that co-culture of wild-type strains of *C. thermocellum* and *Clostridium thermolacticum* resulted in enhanced ethanol yield (80% of the theoretical maximum) during cellulose fermentation [74]. Another study demonstrated the effectiveness of co-culturing with *C. thermocellum* strain LQRI and *Thermoanaerobacter pseudethanolicus* strain X514, for improved ethanol production during batch fermentation [70]. Furthermore, this co-culture was applied to a semi-continuous cyclic fed-batch fermentor resulting in an impressive ethanol yield of 474 mM in 96 h with an initial higher cellulose concentration of 80 g L^−1^, under pH-controlled conditions [75].

### Co-culture fermentation of rice straw biomass (RSB)

Co-cultivation was found to be effective on model substrates; thus these co-culture combinations were further incubated with native and deacetylated dilute acid-pretreated RSB to test their effectiveness on real substrates. On pretreated RSB at 96 h of fermentation, the ethanol yield by the co-culture reached to 22.13 mM (C19 + DC21), 20.49 mM (C19 + X2), and 25.28 mM (C19 + DC21 + X2) (Fig. 6d), suggesting nearly a two-fold improvement in yield compared to monoculture.

Similar observation on ethanol yield improvement was observed on native RSB as well using these co-culture combinations. The highest ethanol and total soluble metabolite concentration on washed native RSB were 9 and 17.33 mM, respectively, achieved by C19 + DC21 + X2 co-culture combination (Fig. 6e). This represents nearly 3 times improvement in ethanol yields achieved by the co-culture than the monoculture strain DBT-IOC-C19 (2.51 mM) and strain DBT-IOC-DC21 (2.00 mM) alone.

Previous co-culture studies demonstrating effectiveness of high-temperature CBP to produce ethanol and hydrogen from a variety of low-cost-renewable feedstocks such as alkali-treated corn cob [58], alkali-treated sugar cane bagasse [76], straw–hay mixture [24], and dilute acid-pretreated poplar and Miscanthus [77] have been attempted. However, this study represents the maiden report on the co-culture cultivation on deacetylated dilute acid-pretreated RSB as substrate with the purpose of cellulosic ethanol production.

Although co-culture showed improved performance over individual strains, mixed acid fermentation of substrates by these strains is complicated. Moreover, if the hydrolysis and fermentation performance can be further improved, an effective process could be developed for sustainable and cost-effective conversion of RSB to ethanol, suggesting their suitability for CBP approach.

## Conclusions

In this study, a novel thermophilic bacterium *Clostridium* sp. DBT-IOC-C19 isolated from a hot spring effectively utilized the lignocellulosic biomass for ethanol production in a single step. Initial substrate concentration and pH were identified as important factors controlling the fermentation profile of this strain. Co-culture combinations of novel thermophilic anaerobic bacterium improved total soluble metabolite concentration compared to monoculture. The ethanol yields produced by the wild-type strain in the present study are encouraging, though not at industrially relevant levels as yet. However, the strain clearly displayed single-step conversion of biomass that indicates its suitability for CBP approach.

## Methods

### Reagents and chemicals

All chemicals used in this study were of analytical grade obtained from Sigma-Aldrich, (Bengaluru, KA, India), Himedia (India), and Merck Limited (New Delhi, India). For DNA isolation and purification, molecular biology-grade chemicals were used. The DNeasy Blood & Tissue Kit, QIA quick Gel Extraction Kit, and Hot Star Taq Master Mix Kit were obtained from Qiagen India Pvt. Ltd., (New Delhi, India). All gases used were purchased from Inox air products limited, India.

### Sampling site

Samples were collected from the Puga thermal hot spring (N 33°13′23″; E 78°19′12″ altitude 4400 m), located in the Indus valley of eastern Ladakh region of North West Himalaya, Jammu & Kashmir, India. The surface temperature of geothermal water was around 82 °C with slightly alkaline pH. Samples were collected anaerobically in serum bottles and transported to laboratory without temperature control.

### Bacterial strains and growth medium

The cellulolytic thermophilic anaerobic bacteria used in this study were isolated from natural hot water and bacterial mat samples collected from Puga thermal hot spring. Enrichment and cultivation were conducted at 60 °C in minimal medium (M), prepared as previously described [78].

The other two strains used in this study, *Clostridium* sp. strain DBT-IOC-DC21 (gene bank accession number; KX842077) and *Thermoanaerobacter* sp. strain DBT-IOC-X2 (gene bank accession number; KY056821), were originally isolated from the compost and hot spring site, respectively, and maintained in the laboratory. Strain DBT-IOC-DC21 is predominantly xylanolytic, while strain DBT-IOC-X2 is non-cellulolytic but sugar-fermenting. Both strains were thermophilic and cultured at 70 °C on an M medium containing 10 g L^−1^ glucose unless stated otherwise. Inoculum for fermentation experiments was prepared from a single colony of isolate that was maintained at −80 °C in 2 mL aliquots containing 30% deoxygenated glycerol.

*Clostridium thermocellum* strain DSM 1313 was procured from the DSMZ collection (Deutsche Sammlung von Mikroorganismen und Zellkulturen GmbH). A single colony from the grown culture was stored in 30% deoxygenated glycerol at −80 °C. After transferred thrice on MTC medium [33, 79] with 10 g L^−1^ Avicel PH-101 as the carbon source, grown culture was used as 5% (v/v) inoculum for the fermentation experiments.

Standard anaerobic culture techniques as proposed by Hungate [80] and modified by Bryant [81] were used throughout this study. All the media used in this study were prepared by boiling with a constant flow of nitrogen gas, cooled, and dispensed in the serum bottles inside a Coy anaerobic chamber (USA) with a headspace of N_2_: CO_2_: H_2_ (90:5:5). Ten milliliter media was used in anaerobic Hungate culture tubes (16 × 125 mm, Bellco glass) and 50 mL in the serum bottles (125 mL, Wheaton). Serum bottles were sealed with butyl rubber stoppers (Bellco, USA) and aluminum crimp seals. A stock solution of l-cysteine HCl was used as a reducing agent for the preparation of pre-reduced medium for all growth and fermentation studies. Concentrated stocks of sugar and vitamin solutions were prepared separately and filter sterilized into preautoclaved anaerobic bottles. Desired concentration of these stock solutions was added to the media bottles just before inoculation. After mixing all the solutions, final pH of the media was adjusted to pH 8.0 via addition of 1 M sodium hydroxide solution, unless stated otherwise.

### Enrichment and isolation

The hot spring samples were freshly inoculated into anaerobic M medium (120 mL, pH 7.0) containing 10 g L^−1^ Whatman No. 1 filter paper as the sole carbon source to enrich cellulose-degrading thermophilic anaerobic bacteria. The cultures were incubated at 60 °C, as the suggestive temperature optima for most *C. thermocellum* strains [8], without shaking till a visible degradation of filter paper were observed. The positive enrichment cultures were inoculated into fresh enrichment medium containing 15 g L^−1^ pretreated rice straw (dry weight basis) and cultured for other 4–5 days. To attempt isolation, first stable microbial consortia were established after about five consecutive transfers in the same medium. Individual colonies were purified from stable enrichment cultures using Hungate roll tube technique. Briefly, serially diluted samples were plated on solidified M medium (3% w/v agar) containing 5 g L^−1^ phosphoric acid-swollen amorphous cellulose [82] in a roll tube [80] and incubated at 50 °C (to avoid melting of agar) in an upright position. The agar block containing well-formed colonies was transferred to fresh liquid medium, inside anaerobic chamber. The purification was repeated several times till a single colony was observed. The isolated cellulolytic bacteria were then grown sequentially on 5 g L^−1^ cellulose- and cellobiose-containing medium several times to ensure the purity of isolated colonies. Further the purity of each isolates was confirmed by 16S rRNA gene sequencing. The grown culture of purified isolates was stored as 2-mL aliquots 30% deoxygenated glycerol at −80 °C and revived before each experiment.

### Strain identification

The genomic DNA of the isolates was extracted using a DNeasy blood and tissue kit (Qiagen Pvt. Ltd) following the manufacturer’s instructions. DNA was then amplified by polymerase chain reaction (PCR) on a Thermal cycler (Applied Biosystems Gene Amp^®^ PCR system 9700, Life Technologies) employing bacterial domain-specific primers for 16S rRNA: 27F 5′-AGAGTTTGATCMTGGCTCAG-3′ and 1492R 5′-CGGTTACCTTGTTACGACTT-3′ [83]. A 20-µL PCR reaction mixture contained 10-µL of Hot Star Taq Master Mix (ready to use solution containing Taq DNA polymerase, dNTPs, MgCl2, Qiagen) and 0.2-µL each of forward and reverse primers to amplify 2-µL of genomic DNA template. The conditions for the PCR amplification were as follows: initial denaturation (5 min at 95 °C) with annealing (1 min at 55 °C) and extension at 72 °C for 1 min followed by final extension at 72 °C for 10 min. PCR was run for 35 cycles. The amplified DNA was purified from agarose gel using the QIA quick Gel Extraction Kit (Qiagen India Pvt. Ltd., India) and sent for sequencing to Institute of Microbial Technology (Chandigarh, India).

The 16S rRNA gene sequences of the strains were compared with the sequences of type strains available in the GenBank database using NCBI BLAST server (http://blast.ncbi.nlm.nih.gov/Blast/; National Centre for Biotechnology Information, MD, USA). Sequences were aligned using CLUSTAL W and phylogenetic tree was constructed according to the Neighbor-Joining method [84] using MEGA 7.0 software [85]. The percentage of replicate trees in which the associated taxa clustered together by bootstrap test for 1000 replicates to estimate the confidence of branching [86]. The evolutionary distances were computed using the Maximum Composite Likelihood method [87]. The 16S rRNA gene sequence of strain DBT-IOC-C2, DBT-IOC-C15, and DBT-IOC-C19 are submitted to GenBank with nucleotide accession numbers KX842074, KX842075, and KX842076, respectively.

### Growth optimization

Media with different initial pH values in the range from 3.0 to 10.0 at intervals of 1 unit was adjusted by supplementing 1 M HCL and 1 M NaOH solution. The optimum pH was determined first at the experimental temperature (i.e., 60 °C) followed by incubation at different temperatures increased gradually from 45 to 85 °C at 5 °C intervals at selected pH values to determine optimal temperature for growth. Strains were adapted by transferring at least twice to the new conditions before performing each measurement. All growth optimization experiments were performed in triplicate in serum bottles containing 50 mL M medium with 10 g L^−1^ cellobiose, as the sole carbon source. Inoculum of freshly grown culture (OD_600_ ~0.8–1.0) prepared by passaging thrice on M medium was inoculated (5%, v/v) and incubated for 24 h without shaking. Cell growth was inferred by measuring optical density at 600 nm (OD_600_) at the beginning and at the end of incubation time. The cell growth was inferred by measuring optical density at 600 nm (OD_600_). The optimized pH and temperature condition determined were thereafter used in all experiments performed.

### Cross substrate utilization

Multiple substrate utilization ability of the isolated strains was tested using various soluble (cellobiose, glucose, xylose, arabinose, mannose, galactose, fructose, maltose, lactose, carboxy methyl cellulose sodium salt, and sucrose) and insoluble (starch, Whatman no. 1 filter paper, xylan from oat spelt, microcrystalline cellulose; Avicel PH-101, washed native and deacetylated dilute acid-pretreated rice straw) carbon sources supplemented to 10 mL M medium and incubated under respective optimum conditions for 24–96 h.

The growth on soluble substrates was monitored by measuring the optical density of the cultures (OD_600_) at the beginning and at the end of incubation, while on insoluble substrates analysis of fermentation products was performed. For the separation of cells from insoluble substrates, samples were centrifuged in 1.5 mL centrifuge tube at 7000 rpm for 5 min. For each substrate, triplicate reactions and a control without inoculation were included. Strict anaerobic techniques were followed throughout the experimental manipulations.

### Batch fermentation studies

All batch fermentations were performed in 125-mL serum bottles with 50 mL of MTC medium supplemented with different substrates such as simple sugars (glucose and cellobiose), complex polysaccharides (cellulose, xylan, and their mixture), and lignocellulosic biomass (native and dilute acid-pretreated rice straw) at a final concentration of 5 g L^−1^, unless otherwise specified. The influence of varying cellulose concentrations from 5 to 60 g L^−1^ in 125 mL serum bottles was investigated in MTC medium (initial pH 8.0).

Each experiment set was inoculated with inoculum from freshly grown culture (OD_600_ ~0.8–1.0) prepared by passaging thrice on MTC medium containing 10 g L^−1^ cellobiose as the sole carbon source, unless otherwise specified. In monoculture studies, the inoculation ratio was fixed to 5% (v/v) of the medium. For the initiation of co-culture, monoculture of each strain was passaged at least two times on the same medium containing cellobiose, prior to the start of fermentation studies. The co-culture set was inoculated with 2.5% (v/v) of each inoculum for the co-culture of two strains and 1.64% (v/v) of each inoculum for the co-culture of three strains. Incubations were conducted anaerobically at 60 °C under static conditions and a starting pH 7.5. Well-mixed samples were collected at specified time intervals for analysis. All fermentations were performed in triplicate and values are reported as mean along with the standard deviation and statistical analysis.

### Preparation of dilute acid-treated rice straw in the pilot plant and its composition analysis

Rice straw (*Oryza sativa*) was used as the lignocellulosic substrate for fermentation studies and was collected from local market (Mathura district, Uttar Pradesh, India) [3]. Rice straw was air-dried and milled to 4–5 mm particle size using a milling machine for pretreatment (Texol, Pune, India) and stored in air-sealed containers at 25 °C until further use. Dilute acid pretreatment of rice straw was performed in a 250 kg biomass per day capacity continuous pilot scale plant capable of operating with multiple feedstocks under a wide range of operating conditions [88]. Air-dried rice straw was soaked in sulfuric acid solution at 1% (w/w) concentration followed by alkali soaking at 0.4% (w/w) concentration in a soaking chamber specially equipped with spray and circulation of acid solution. The wet biomass, after soaking was hung for 2 h and further pressed for 15 min at a pressure up to 100 bars in a hydraulic filter press to remove water. This biomass was subjected to pretreatment in the reactor at temperature 162 °C, pressure 5 bar, and residence time of 10 min. Residence time was controlled by the screw speed of the reactor. The pretreated biomass slurry was collected in the slurry tank, cooled, and then transferred through a peristaltic pump to a high speed centrifuge for separating solids (cellulose and lignin) and liquid (hemicelluloses) fractions. The solid portion of pretreated rice straw, after washing with deionized water, was used for this study. The composition of native and pretreated rice straw was determined according to the laboratory analytical procedure “Determination of structural carbohydrates and lignin in biomass” from National Renewable Energy Laboratory (NREL) [68]. The solid residual biomass was stored at −20 °C until further use. All the experiments were conducted using a single lot of these substrates.

### Analytical methods

End products of fermentation were analyzed for metabolites (lactate and acetate) and residual carbohydrates (glucose, cellobiose, xylose, and arabinose) using high-performance liquid chromatograph (HPLC) (Waters Corp. USA) equipped with Bio-Rad Aminex HPX-87H column (Bio-Rad Laboratories, USA), refractive index detector (RID), and UV detector and operated using 0.05 M H2SO4 as the mobile phase (flow rate, 0.6 mL min^−1^; column temperature, 50 °C) [88]. Identification of peaks was performed by comparison of retention times with standards area.

Ethanol was analyzed using Clarus-680 Gas Chromatograph (Perkin-Elmer, USA) fitted with a 30-m-long Stabilwax^®^-DA column (Restek) having an inner diameter of 0.25 mm [89]. Gas chromatography was operated at an initial oven temperature of 50 °C for 5 min, followed by 120 °C for 5 min with a ramp rate of 15 °C per min. Helium gas, at a flow rate of 2 mL min^−1^, was used as carrier and 2-Propanol was used as the internal standard. All samples were appropriately diluted and filtered through a 0.2-μm filter before each chromatographic analysis.

The moisture content of cellulosic substrates was determined according to NREL LAP [68] using an infrared drier from Sartorius MA-150C (Model No. 000230V1), Germany. Compositional analysis of native and pretreated rice straw was carried out by two-stage acid hydrolysis following the NREL/TP-510-42618 and HPLC method NREL/TP-510-42623 [69]. Briefly, the samples were analyzed for carbohydrate composition using a HPLC (Waters, USA) equipped with a refractive index detector and UV–Vis detector. The analysis of carbohydrates (glucose, xylose, galactose, mannose, and arabinose) was performed using Aminex HPX-87P column (Biorad, USA) coupled with refractive index detector. The mobile phase was milli-Q water at a flow rate of 0.6 mL min^−1^, with a column temperature of 75 °C and inhibitor products (furfural and 5-hydroxy methyl furfural) were separated using Aminex HPX-87H column (Biorad, USA) coupled with UV–Vis detector. The mobile phase was 0.008 N H_2_SO_4_ at a flow rate of 0.6 mL min^−1^, with a column temperature of 50 °C. Both the columns were equipped with suitable guard columns. Residual cellulose concentration was determined on the basis of gravimetric analysis as described previously [90].

$${\text{Cellulose conversion }}\left( \% \right) = \frac{{{\text{Amount of cellulose added}} - {\text{ Residual cellulose}}}}{\text{Amount of cellulose added}} \times 100$$

### Statistical analysis

Statistical significance among groups was determined by using the one-way analysis of variance (ANOVA) followed by Tukey’s honest significant difference (HSD) post hoc tests. For statistical analysis, the product concentrations in triplicate cultures were compared. Details of experimental design are described in text. All results are expressed as average ±  standard deviation and differences considered significant at probability value less than 0.05 (*p* < 0.05). Statistical analyses were performed using R-Studio^®^, version 1.0.136 (RStudio, Inc. Boston, MA).

## Additional file

**Additional file 1: Table S1.** Fermentation products from Whatman No. 1 filter paper and pretreated rice straw biomass during enrichment cultivation. **Table S2.** Fermentation products of 19 cellulose-degrading thermophilic anaerobic bacteria on crystalline cellulose. **Figure S1.** Growth optimization for pH and temperature conditions. **Figure S2.** Batch fermentation on ‘Minimal media for thermophilic clostridia’. **Table S3.** Cross substrate utilization test. **Table S4.** Time course of crystalline cellulose consumption by *Clostridium* sp. strains DBT-IOC-C19. **Table S5.** Comparative fermentation performance of *Clostridium* sp. strain DBT-IOC-C19 and *Clostridium thermocellum* DSM 1313. **Table S6.** Co-culture fermentation performance.
