# Supplementary material for: Cellulosic ethanol production via consolidated bioprocessing by a novel thermophilic anaerobic bacterium isolated from a Himalayan hot spring
Source: Biotechnol Biofuels. 2017 Mar 21;10:73. doi: 10.1186/s13068-017-0756-6 (PMC5361838; doi:10.1186/s13068-017-0756-6)
Supplement: Supplementary file 1 — Additional file 1: Table S1. Fermentation products from Whatman No. 1 filter paper and pretreated rice straw biomass during enrichment cultivation. Table S2. Fermentation products of 19 cellulose-degrading thermophilic anaerobic bacteria on crystalline cellulose. Figure S1. Growth optimization for pH and temperature conditions. Figure S2. Batch fermentation on ‘Minimal media for thermophilic clostridia’. Table S3. Cross substrate utilization test. Table S4. Time course of crystalline cellulose consumption by Clostridium sp. strains DBT-IOC-C19. Table S5. Comparative fermentation performance of Clostridium sp. strain DBT-IOC-C19 and Clostridium thermocellum DSM 1313. Table S6. Co-culture fermentation performance. [file 13068_2017_756_MOESM1_ESM.docx]

**Table S1: Fermentation products from filter paper and pretreated rice straw during enrichment cultivation**

| **Enrichment cultures** | **Growth on filter paper (15 g liter^-1^)** | | | | |  | **Growth on pretreated rice straw (15 g liter^-1^ dry weight)** | | | | |
| --- | --- | --- | --- | --- | --- | --- | --- | --- | --- | --- | --- |
|  | **Ethanol (mM)** | **Acetate (mM)** | **Lactate (mM)** | **pH** | **Total products (mM)** |  | **Ethanol (mM)** | **Acetate (mM)** | **Lactate (mM)** | **pH** | **Total products (mM)** |
| #1 | 37.88 ± 0.34 | 16.40 ± 1.73 | 6.05 ± 0.55 | 5.63 ± 0.04 | 60.32 ± 2.62 |  | 16.84 ± 0.12 | 10.71 ± 0.11 | 2.64 ± 0.12 | 5.83 ± 0.03 | 30.20 ± 0.35 |
| #2 | 29.60 ± 0.26 | 24.39 ± 0.72 | 3.82 ± 0.52 | 5.63 ± 0.02 | 57.80 ± 1.49 |  | 11.27 ± 0.52 | 8.61 ± 0.07 | 1.21 ± 0.05 | 6.33 ± 0.03 | 21.09 ± 0.64 |
| #3 | 33.84 ± 1.17 | 16.49 ± 1.61 | 6.73 ± 0.26 | 5.62 ± 0.02 | 57.06 ± 3.04 |  | 9.33 ± 0.61 | 8.17 ± 0.04 | 3.39 ± 0.11 | 6.37 ± 0.01 | 20.89 ± 0.76 |
| #4 | 22.66 ± 0.74 | 21.94 ± 0.29 | 4.94 ± 0.39 | 5.71 ± 0.01 | 49.54 ± 1.42 |  | 20.21 ± 0.18 | 9.31 ± 0.14 | 2.40 ± 0.09 | 5.83 ± 0.01 | 31.92 ± 0.41 |
| #5 | 31.21 ± 0.21 | 17.87 ± 1.18 | 3.95 ± 0.07 | 5.69 ± 0.00 | 53.03 ± 1.46 |  | 18.74 ± 0.91 | 11.08 ± 0.06 | 1.59 ± 0.09 | 5.83 ± 0.05 | 31.42 ± 1.06 |
| #6 | 34.14 ± 0.58 | 25.48 ± 0.24 | 4.96 ± 0.71 | 5.60 ± 0.00 | 64.59 ± 1.53 |  | 16.77 ± 0.17 | 11.85 ± 0.06 | 3.57 ± 0.05 | 5.80 ± 0.01 | 32.18 ± 0.27 |

Enrichment cultures were selected on filter paper and pretreated rice straw from thermal hot spring samples.

Cultivations were performed in 250 ml serum bottle containing 120 ml M medium at 60°C and at initial pH of 7.0

Results are presented as mean ± standard deviation of samples prepared in duplicate.

Uninoculated controls for both the substrates tested were analyzed to considered non-biological fermentation.

**Table S2** **Fermentation products of 19 cellulose-degrading thermophilic anaerobic bacteria on crystalline cellulose**

| **Enrichment culture used for isolation** | **Isolates** | **Fermentation products (mM)** | | | |
| --- | --- | --- | --- | --- | --- |
|  |  | **Ethanol (mM)** | **Lactate (mM)** | **Acetate (mM)** | **Total products (mM)** |
| #1 | DBT-IOC-C1 | 12.52 ± 0.16 | 0.63 ± 0.05 | 4.53 ± 0.16 | 17.68 ± 0.37 |
| #1 | DBT-IOC-C2 | 15.80 ± 0.08 | 2.67 ± 0.12 | 8.15 ± 0.13 | 26.62 ± 0.32 |
| #5 | DBT-IOC-C3 | 0.85 ± 0.07 | 0.47 ± 0.10 | 7.92 ± 0.16 | 9.24 ± 0.33 |
| #2 | DBT-IOC-C4 | 10.66 ± 0.23 | 0.32 ± 0.06 | 4.20 ± 0.10 | 15.18 ±0.38 |
| #2 | DBT-IOC-C5 | 12.20 ± 0.08 | 0.90 ± 0.05 | 3.81 ± 0.12 | 16.91 ± 0.24 |
| #2 | DBT-IOC-C6 | 4.93 ± 0.12 | 2.12 ± 0.06 | 2.64 ± 0.08 | 9.68 ± 0.26 |
| #3 | DBT-IOC-C7 | 8.02 ± 0.13 | 0.79 ± 0.08 | 4.28 ± 0.26 | 13.08 ± 0.47 |
| #4 | DBT-IOC-C8 | 9.64 ± 0.16 | 0.90 ± 0.08 | 3.94 ± 0.12 | 14.48 ± 0.36 |
| #6 | DBT-IOC-C9 | 11.01 ± 0.25 | 0.29 ± 0.05 | 8.46 ± 0.14 | 19.76 ± 0.44 |
| #5 | DBT-IOC-C10 | 5.58 ± 0.14 | 0.66 ± 0.06 | 4.41 ± 0.19 | 10.64 ± 0.39 |
| #1 | DBT-IOC-C11 | 11.03 ± 0.22 | 1.14 ± 0.06 | 3.90 ± 0.12 | 16.06 ± 0.39 |
| #2 | DBT-IOC-C12 | 7.25 ± 0.19 | 0.21 ± 0.02 | 1.89 ± 0.05 | 9.34 ± 0.26 |
| #4 | DBT-IOC-C13 | 6.29 ± 0.09 | 0.32 ± 0.06 | 3.46 ± 0.05 | 10.07 ± 0.20 |
| #3 | DBT-IOC-C14 | 9.02 ± 0.19 | 0.66 ± 0.05 | 6.50 ± 0.05 | 16.17 ± 0.30 |
| #3 | DBT-IOC-C15 | 14.27 ± 0.13 | 0.74 ± 0.03 | 7.94 ± 0.10 | 22.94 ± 0.26 |
| #4 | DBT-IOC-C16 | 10.32 ± 0.14 | 0.43 ± 0.07 | 2.10 ± 0.14 | 12.85 ± 0.35 |
| #1 | DBT-IOC-C17 | 8.34 ± 0.12 | 0.42 ± 0.05 | 5.70 ± 0.12 | 14.47 ± 0.29 |
| #2 | DBT-IOC-C18 | 5.66 ± 0.03 | 0.73 ± 0.06 | 4.27 ± 0.14 | 10.65 ± 0.23 |
| #5 | DBT-IOC-C19 | 17.43 ± 0.19 | 1.42 ± 0.03 | 6.41 ± 0.14 | 25.27 ± 0.36 |

Pure culture of cellulose-degrading thermophilic anaerobic bacteria were grown on 10 g liter^-1^ crystalline cellulose in 125 ml serum bottle containing 50 ml defined M medium and incubated at 60ºC and at initial pH 7.0, without shaking.

Fermentation of cellulose by uninoculated controls was less than 10%.

Results are presented as mean ± SD of samples prepared in triplicate.

**Figure S1** **Growth optimization for pH and temperature conditions**

**(A) *Clostridium sp.* strain DBT-IOC-C2**

**(B) *Clostridium sp.* strain DBT-IOC-C15**

**(C) *Clostridium sp. s*train DBT-IOC-C19**

The strains were cultured at various temperature and pH for 24 h as static cultures in closed 125 ml serum bottle (50 ml M medium)

containing 10 g liter^-1^ cellobiose. Growth was monitored by measuring optical density (OD) at 600 nm and expressed as actual OD of the

sample minus the OD of uninoculated control samples.

**Figure S2** **Batch fermentation of thermophilic clostridia on the ‘Minimal media’**

**Fermentation profile of *Clostridium sp.* strain DBT-IOC-C19, *Clostridium sp.* strain DBT-IOC-C15 and *Clostridium sp.* strain DBT-IOC-C2 for product formation on MTC medium with 10 g liter^-1^ crystalline cellulose, under optimized conditions. Each data point represents the average and ± standard deviation calculated from triplicate fermentations sampled at 96 h.**

**Table S3** **Cross substrate utilization test**

| **Substrate** | **DBT-IOC-C2** | **DBT-IOC-C15** | **DBT-IOC-C19** |
| --- | --- | --- | --- |
| **Glucose** | **+** | **+** | **+** |
| **Xylose** | **-** | **-** | **-** |
| **Cellobiose** | **+** | **+** | **+** |
| **Arabinose** | **+** | **-** | **+** |
| **Mannose** | **-** | **-** | **-** |
| **Galactose** | **+** | **+** | **+** |
| **Fructose** | **-** | **-** | **-** |
| **Maltose** | **-** | **-** | **-** |
| **Lactose** | **-** | **-** | **-** |
| **Sucrose** | **-** | **-** | **-** |
| **Starch** | **~** | **-** | **~** |
| **Carboxy methyl cellulose** | **+** | **+** | **+** |
| **Filter paper** | **+** | **+** | **+** |
| **Avicel** | **+** | **+** | **+** |
| **Xylan (Oat spelt)** | **+** | **+** | **+** |
| **Pretreated rice straw** | **+** | **+** | **+** |
| **Untreated rice straw** | **+** | **+** | **+** |

**Table S4** **Time course of crystalline cellulose consumption by *Clostridium sp. s*trains DBT-IOC-C19**

**(A). Fermentation products of *Clostridium sp. s*trains DBT-IOC-C19 at different cellulose concentrations**

| **Avicel**  **(g liter^-1^)** | **Fermentation products (mM)** | | | | |
| --- | --- | --- | --- | --- | --- |
|  | **Ethanol (mM)** | **Lactate (mM)** | **Acetate (mM)** | **pH** | **Total products (mM)** |
| 5 | 22.87 ± 1.64 | 3.33 ± 0.11 | 8.73 ± 0.17 | 5.98 ± 0.03 | 34.92 ± 1.92 |
| 10 | 34.51 ± 1.32 | 4.74 ± 0.11 | 17.40 ± 0.16 | 5.72 ± 0.02 | 56.65 ± 1.60 |
| 20 | 37.09 ± 0.05 | 5.48 ± 0.06 | 21.24 ± 0.18 | 5.70 ± 0.02 | 63.82 ± 0.30 |
| 30 | 33.63 ± 0.61 | 4.93 ± 0.08 | 20.23 ± 0.18 | 5.78 ± 0.02 | 58.79 ± 0.88 |
| 40 | 15.80 ± 1.28 | 5.19 ± 0.08 | 14.12 ± 0.20 | 6.00 ± 0.05 | 35.10 ± 1.50 |
| 50 | 12.22 ± 0.47 | 5.00 ± 0.11 | 6.36 ± 0.07 | 6.67 ± 0.05 | 23.58 ± 0.74 |
| 60 | 10.67 ± 0.30 | 4.09 ± 0.07 | 5.85 ± 0.08 | 6.74 ± 0.04 | 20.61 ± 0.45 |

Cultivations were performed in 125 ml serum bottle containing 50 ml MTC medium with various Avicel concentrations and incubated at 60ºC and at initial pH 7.5 without shaking.

Data obtained after 96 h of fermentation are presented as mean ± standard deviation of samples prepared in triplicate. Fermentation of Avicel by uninoculated controls was less than 10%.

**(B). Time course of crystalline cellulose consumption by *Clostridium sp. s*trains DBT-IOC-C19 at different cellulose concentrations**

| **Time (hour)** | **5 g liter^-1^** | **10 g liter^-1^** | **20 g liter^-1^** | **30 g liter^-1^** | **40 g liter^-1^** | **50 g liter^-1^** | **60 g liter^-1^** |
| --- | --- | --- | --- | --- | --- | --- | --- |
| 0 | 4.94 ± 0.07 | 9.97 ± 0.04 | 20.06 ± 0.10 | 30.07 ± 0.19 | 39.98 ± 0.20 | 50.17 ± 0.92 | 60.64 ± 0.58 |
| 24 | 4.09 ± 0.12 | 7.48 ± 0.12 | 18.05 ± 0.27 | 28.00 ± 0.19 | 39.67 ± 0.06 | 48.20 ± 0.86 | 59.29 ± 0.14 |
| 48 | 3.50 ± 0.11 | 4.59 ± 0.04 | 15.06 ± 0.32 | 26.95 ± 0.28 | 38.44 ± 0.08 | 47.05 ± 0.32 | 59.81 ± 0.18 |
| 72 | 1.02 ± 0.03 | 3.42 ± 0.07 | 11.22 ± 0.16 | 25.14 ± 0.18 | 37.08 ± 0.18 | 47.18 ± 0.24 | 58.03 ± 0.17 |
| 96 | 0.82 ± 0.07 | 1.95 ± 0.08 | 11.12 ± 0.08 | 22.19 ± 0.12 | 35.10 ± 0.20 | 45.94 ± 0.58 | 56.32 ± 0.07 |
| 120 | 0.47 ± 0.07 | 1.85 ± 0.09 | 11.14 ± 0.12 | 22.11 ± 0.27 | 34.69 ± 0.24 | 45.77 ± 0.45 | 56.45 ± 0.17 |
| 144 | 0.27 ± 0.07 | 1.72 ± 0.05 | 11.03 ± 0.16 | 22.18 ± 0.22 | 34.87 ± 0.08 | 45.97 ± 0.30 | 56.33 ± 0.15 |

Cultivations was performed in 125 ml serum bottle containing 50 ml MTC medium with various Avicel concentrations and incubated at 60ºC and at initial pH 7.5 without shaking.

Data obtained after 96 h of fermentation are presented as mean ± standard deviation of samples prepared in triplicate. Fermentation of Avicel by uninoculated controls was less than 10%.

**(C) Degradation kinetics of cellulose by *Clostridium sp. s*train DBT-IOC-C19**

| **Time (Hour)** | **Fermentation products (mM)** | | | | |
| --- | --- | --- | --- | --- | --- |
|  | **Ethanol (mM)** | **Lactate (mM)** | **Acetate (mM)** | **pH** | **Total products (mM)** |
| 0 | 0.00 ± 0.00 | 0.00 ± 0.00 | 0.00 ± 0.00 | 7.53 ± 0.02 | 0.00 ± 0.00 |
| 24 | 7.51 ± 0.51 | 0.12 ± 0.00 | 3.47 ± 0.13 | 7.11 ± 0.02 | 11.09 ± 0.63 |
| 48 | 12.39 ± 0.57 | 1.64 ± 0.13 | 8.04 ± 0.31 | 6.15 ± 0.04 | 22.07 ± 1.01 |
| 72 | 30.77 ± 1.80 | 4.19 ± 0.28 | 15.05 ± 0.70 | 5.85 ± 0.03 | 50.01 ± 2.78 |
| 96 | 34.58 ± 0.64 | 4.88 ± 0.17 | 18.15 ± 0.20 | 5.76 ± 0.02 | 57.61 ± 1.01 |
| 120 | 35.79 ± 1.03 | 4.81 ± 0.16 | 18.50 ± 1.58 | 5.74 ± 0.02 | 59.10 ± 2.77 |
| 144 | 34.85 ± 0.78 | 4.80 ± 0.22 | 17.99 ± 1.03 | 5.73 ± 0.03 | 57.64 ± 2.03 |

Experiments were performed as described in the text.

Values are presented as mean ± standard deviation of the samples prepared in triplicate.

Fermentation of Avicel by uninoculated controls was less than 10%.

**Table S4** **The comparison of fermentation performance between *Clostridium sp.* DBT-IOC-C19 and *Clostridium thermocellum* DSM 1313**

| **Strains** | **Substrate** | **Fermentation products (mM)** | | | | |
| --- | --- | --- | --- | --- | --- | --- |
|  |  | **Ethanol (mM)** | **Lactate (mM)** | **Acetate (mM)** | **pH** | **Total products (mM)** |
| Strain DBT-IOC-C19 | Glucose (55.5 mM) | 20.42 ± 0.04 | 3.54 ± 0.13 | 15.97 ± 0.25 | 5.95 ± 0.01 | 39.93 ± 0.42 |
|  | Cellobiose (31 mM) | 26.55 ± 1.22 | 4.02 ± 0.07 | 16.74 ± 0.27 | 5.85 ± 0.02 | 47.31 ± 1.56 |
|  | Avicel (61.67 mM glucose equiv.) | 32.55 ± 0.29 | 5.14 ± 0.04 | 18.67 ± 0.28 | 5.77± 0.03 | 56.36 ± 0.61 |
|  | Xylan (58.6 mM xylose equiv.) | 11.45 ± 0.94 | 2.04 ± 0.26 | 4.46 ± 0.06 | 6.56± 0.03 | 17.95 ± 1.26 |
|  | Pretreated rice straw (61.67 mM glucose equiv.) | 14.15 ± 0.26 | 2.31 ± 0.14 | 9.05 ± 0.31 | 6.24 ± 0.04 | 25.52 ± 0.71 |
|  | Untreated rice straw (61.67 mM glucose equiv.) | 2.52 ± 0.14 | 0.92 ± 0.02 | 1.88 ± 0.04 | 7.35 ± 0.05 | 5.31 ± 0.20 |
|  | | | | | | |
| Strain DSM 1313 | Glucose (55.5 mM) | 25.12 ± 0.54 | 2.74 ± 0.19 | 18.08 ± 0.27 | 5.84 ± 0.03 | 45.95 ± 1.00 |
|  | Cellobiose (31 mM) | 32.93 ± 0.12 | 2.82 ± 0.06 | 20.77 ± 0.79 | 5.76 ± 0.01 | 56.52 ± 0.97 |
|  | Avicel (61.67 mM glucose equiv.) | 37.35 ± 0.28 | 3.03 ± 0.05 | 22.35 ± 0.68 | 5.71 ± 0.02 | 62.73 ± 1.01 |
|  | Xylan (58.6 mM xylose equiv.) | 10.23 ± 0.53 | 1.21 ± 0.11 | 7.28 ± 0.03 | 6.62 ± 0.02 | 18.72 ± 0.68 |
|  | Pretreated rice straw (61.67 mM glucose equiv.) | 11.93 ± 0.21 | 1.45 ± 0.03 | 10.72 ± 0.10 | 6.41 ± 0.04 | 24.09 ± 0.34 |
|  | Untreated rice straw (61.67 mM glucose equiv.) | 5.23 ± 0.02 | 0.48 ± 0.06 | 3.99 ± 0.02 | 7.16 ± 0.04 | 9.70 ± 0.10 |

Cultivations were performed on 50 ml MTC medium at 60°C; pH 7.5 (for strain DBT-IOC-C19) and pH 7.0 (for strain DSM 1313), for 96 h in 125 ml serum bottles without shaking.

Results are presented as mean ± standard deviation of the samples prepared in triplicate.

All substrates were loaded at equivalent concentration expressed as xylose and hexose equivalents, after moisture correction.

Fermentation of various substrates by uninoculated duplicate controls was less than 10%.

**Table S6** **Co-culture fermentation performance**

| **Co-culture combinations** | **Substrates** | **Fermentation products (mM)** | | | | |
| --- | --- | --- | --- | --- | --- | --- |
|  |  | **Ethanol (mM)** | **Lactate (mM)** | **Acetate (mM)** | **pH** | **Total products (mM)** |
| C19+ DC21 | Avicel (61.67 mM glucose equiv.) | 35.26 ± 0.75 | 5.13 ± 0.05 | 19.62 ± 0.16 | 5.74 ± 0.01 | 60.01 ± 0.96 |
| C19+X2 |  | 36.81 ± 0.23 | 5.87 ± 0.07 | 15.99 ± 0.36 | 5.77 ± 0.01 | 58.67 ± 0.66 |
| C19+DC21+X2 |  | 41.94 ± 0.67 | 4.83 ± 0.06 | 15.22 ± 0.22 | 5.77 ± 0.03 | 61.99 ± 0.95 |
|  | | | | | | |
| C19+ DC21 | Xylan (58.6 mM xylose equiv.) | 17.52 ± 0.47 | 6.48 ± 0.04 | 11.69 ± 0.14 | 5.93 ± 0.03 | 35.69 ± 0.64 |
| C19+X2 |  | 11.42 ± 0.24 | 2.18 ± 0.06 | 4.83 ± 0.12 | 6.75 ± 0.04 | 18.43 ± 0.41 |
| C19+DC21+X2 |  | 23.76 ± 0.87 | 5.73 ± 0.03 | 16.05 ± 0.08 | 5.91 ± 0.02 | 45.54 ± 0.97 |
|  | | | | | | |
| C19+ DC21 | Avicel (30.83 mM glucose equiv.) + Xylan (29.3 mM xylose equiv.) | 37.74 ± 0.46 | 5.54 ± 0.06 | 14.62 ± 0.12 | 5.78 ± 0.02 | 57.91 ± 0.64 |
| C19+X2 |  | 36.95 ± 0.81 | 5.07 ± 0.06 | 13.79 ± 0.18 | 5.78 ± 0.02 | 55.81 ± 1.04 |
| C19+DC21+X2 |  | 38.90 ± 0.66 | 5.59 ± 0.06 | 15.09 ± 0.16 | 5.75 ± 0.04 | 59.59 ± 0.87 |
|  | | | | | | |
| C19+ DC21 | Pretreated rice straw (61.67 mM glucose equiv.) | 22.13 ± 0.32 | 3.71 ± 0.09 | 11.75 ± 0.27 | 5.92 ± 0.02 | 37.60 ± 0.69 |
| C19+X2 |  | 20.49 ± 0.08 | 3.18 ± 0.04 | 10.37 ± 0.14 | 5.96 ± 0.04 | 34.04 ± 0.27 |
| C19+DC21+X2 |  | 25.28 ± 1.22 | 4.53 ± 0.11 | 10.15 ± 0.37 | 5.93 ± 0.03 | 39.96 ± 1.71 |
|  | | | | | | |
| C19+ DC21 | Untreated rice straw (61.67 mM glucose equiv.) | 5.70 ± 0.31 | 1.79 ± 0.10 | 3.67 ± 0.12 | 7.19 ± 0.01 | 11.16 ± 0.53 |
| C19+X2 |  | 4.71 ± 0.55 | 1.42 ± 0.06 | 5.71 ± 0.41 | 7.13 ± 0.02 | 11.84 ± 1.03 |
| C19+DC21+X2 |  | 8.68 ± 0.19 | 2.28 ± 0.10 | 6.37 ± 0.27 | 6.82 ± 0.03 | 17.33 ± 0.56 |

Experiments were performed as described in the text.

Results are presented as mean ± standard deviation of the samples prepared in triplicate.

All substrates were loaded at equivalent concentration expressed as xylose and hexose equivalents, after moisture correction.

Fermentation of various substrates by uninoculated duplicate controls was less than 10%.
